# Slight Temperature Deviation during a 56-Day Storage Period Does Not Affect the Microbiota of Fresh Vacuum-Packed Pork Loins

**DOI:** 10.3390/foods12081695

**Published:** 2023-04-19

**Authors:** Charlotte Braley, Marie-Lou Gaucher, Philippe Fravalo, Fanie Shedleur-Bourguignon, Jessie Longpré, Alexandre Thibodeau

**Affiliations:** 1 Chaire de Recherche en Salubrité des Viandes (CRSV), Faculté de Médecine Vétérinaire, Université de Montréal, Saint-Hyacinthe, QC J2S 2M2, Canada; 2 Département de Pathologie et Microbiologie, Faculté de Médecine Vétérinaire, Université de Montréal, Saint-Hyacinthe, QC J2S 2M2, Canada; 3 Groupe de Recherche et d’Enseignement en Salubrité Alimentaire (GRESA), Faculté de Médecine Vétérinaire, Université de Montréal, Saint-Hyacinthe, QC J2S 2M2, Canada; 4 Centre de Recherche en Infectiologie Porcine et Avicole (CRIPA), Faculté de Médecine Vétérinaire, Université de Montréal, Saint-Hyacinthe, QC J2S 2M2, Canada; 5 Le Conservatoire National des Arts et Métiers (CNAM), 75003 Paris, France; 6 F. Ménard, Division d’Olymel s.e.c., Ange-Gardien, QC J0E 1E0, Canada

**Keywords:** vacuum-packed loin, pork microbiota, storage temperature, temperature deviation, batch production

## Abstract

It is profitable to export fresh meat overseas, where it is often regarded as a premium commodity. Meeting this demand for fresh meat, however, necessitates long export times, during which uncontrolled temperature increases can affect the microbiological quality of the meat and thereby, reduce shelf life or compromise food safety. To study the impact of temperature deviations on microbial community composition and diversity, we used 16S rRNA gene sequencing for *Listeria monocytogenes* and *Salmonella* spp. detection to describe the surface microbiota of eight batches of vacuum-packed loins stored at −1.5 °C (control) for 56 days and subjected to a 2 °C or 10 °C temperature deviation for a few hours (mimicking problems regularly encountered in the industry) at day 15 or 29. The presence of pathogens was negligible. The applied temperature deviations were not associated with different microbiota. Sequencing analysis showed the presence of *Yersinia*, an unexpected pathogen, and relative abundance increased in the groups subjected to temperature deviations. Over time, *Lactobacillales_unclassified* genus became the main constituent of the microbiota of vacuum-packed pork loins. Although the microbiota of the eight batches appeared similar at the beginning of storage, differences were revealed after 56 days, suggesting unequal aging of the microbiota.

## 1. Introduction

In 2021, world pig meat production reached more than 122 million tons. In this global market, Canada exported approximately 1.49 million metric tons of pig meat [1] to more than 90 countries, with China being the largest export market for Canadian pork meat products. The Asian market (China and Japan) was 41% of Canada’s exports, which represents a CAD 4 billion dollar market [2]. Since fresh pork meat products are considered premium commodities by consumers, exporting fresh meat to overseas markets is more profitable than shipping frozen goods. However, maintaining constant control over product temperature for such a long period of time remains a challenge since the organoleptic and microbiological quality of these fresh meat products can be hampered by the increase in storage temperature during long exports [3,4,5].

To limit this problem, most of this exported fresh pork meat is shipped to remote destinations at temperatures just above the freezing point of meat (−1.5 ± 0.5 °C) [6,7]. This extends the product shelf life as much as four times compared to storage at 4 °C [8]. To further increase shelf life, fresh pork meat is packaged under vacuum. Vacuum packaging is a well-documented method for enhancing fresh food preservation. It improves flavor, juiciness, and tenderness of meats [9]. Oxygen removal coupled with storage at subzero temperatures has proven to be very effective at slowing bacterial growth and thereby reducing and slowing spoilage, thus extending product shelf life [10]. 

Spoilage can be defined by a product deterioration beyond consumer acceptance and is mostly the cause of extensive microbial proliferation [5,11]. Aerobic spoilage bacteria such as *Pseudomonas* spp. and *Acinetobacter* spp. were observed to be dominant in refrigerated pork meat products [5,12,13]. Under refrigerated vacuum-packaged conditions, there was a shift from aerobic spoilage bacteria to facultative anaerobic bacteria [14]. Indeed, lactic acid bacteria (LAB) (such as *Lactobacillus* spp., *Lactobacillus curvatus*, *Lactobacillus sakei*, *Leuconostoc* spp., and *Carnobacterium* spp.) and *Brochothrix thermosphacta* were found to be major contributors to meat spoilage and prevailed in pork meat stored at −2 °C [12] or in vacuum-packed pork meat products stored at 4 °C [15]. The spoilage potential of LAB depends on the strain and the quantity of bacteria contaminating the product [5,16]. In the case of overgrowth, LAB produce metabolites such as ethanol and acetic acid, with consequent off-odors, slime, gas formation, and meat discoloration—all typical alterations observable under vacuum packaging [14]. According to several studies [17,18,19,20], the level of LAB contamination, specially *Carnobacterium*, increased over a 20-day storage period under refrigeration. Storage temperatures exceeding 5 °C were correlated with increasing levels of contamination by *Enterobacteriaceae* (such as *Pseudomonas* and *Aeromonas*) [20], clearly showing that storage temperature modified the meat bacterial populations in vacuum packed meat.

The presence of pathogenic bacteria in fresh meat products is also a concern, as they are responsible for human foodborne infections [21]. The bacterial communities of meat products can contain pathogenic bacteria such as *Salmonella* spp. or *Listeria monocytogenes*, bacterial genus that may be present on fresh pork meat products at the end of processing [22,23,24]. *Salmonella* spp. and *Listeria monocytogenes* are able to thrive in food processing environments [23,25]. Vacuum packaging of pork meat has been found to reduce pathogen survival. For example, Djordjević et al. [26] observed that *Salmonella* spp. counts decreased by a reduction of 1.5 log CFU/g during storage under vacuum packaging at refrigerated temperatures. However, it has been reported that *L. monocytogenes*, a psychrotrophic bacterium, was able to grow in vacuum packaged products, especially in ready-to-eat meat [27,28].

Current knowledge on the microbial populations that develop during storage under different vacuum packaging conditions were mainly generated using culture-dependent methods [15,17,29,30,31]. These methods were not able to describe the entire composition of the microbial community. Modern molecular techniques, such as 16S rRNA amplicon sequencing, allow for a deeper characterization of the microbiota using a single analysis and are especially useful for characterizing the presence of bacteria for which culture is not suitable [32]. A few studies used molecular techniques to describe major bacterial populations on fresh pork and the changes that occurred under vacuum packaging conditions throughout a meat processing facility [12,13,15,31,33,34]. Among them, Zhao et al. [15] described the changes in vacuum-packed pork microbiota stored at 0 °C during 21 days. The authors observed that microbiota was more diverse during the first seven days of storage, dominated by the *Microcaccaceae* (27%) and *Flavobacterieaceae* (33%) families, while at the end of storage, the diversity decreased and was dominated by *Lactobacillaceae* (70%), *Enterobacteraceae* (7%), and *Carnobacterium* (4%). Moreover, using sequencing methods, few studies observed that the increase in refrigerated temperature storage influenced the microbiota of vacuum-packed pork meat. For example, Bassey et al. [35] observed that *Rhodococcus* and *Bacillus* dominated in vacuum-packed meat stored at 25 °C, at the beginning and at the end of their experiment (20 days), respectively. However, these studies evaluated the impact of a temperature storage conditions in the whole period of sampling. To our knowledge, no studies described the effect of temperature fluctuations (temporary temperature deviation) on the microbiota of vacuum-packed pork. To our knowledge, no research has described the microbiota evolution of vacuum-packed pork products over a period of several weeks under super chilling conditions that mimic overseas exportation, as well as the impact of early or late temperature deviation on the meat microbiota. Moreover, little is known about microbiota variation between pork meat products originating from different pig batches processed in the same facility during overseas shipping.

The objective of this study was to characterize the impact of temperature deviations on the microbiota of vacuum-packed pork loins according to different production batches. To do so, 16S rRNA amplicon sequencing was used, as well as culture-dependent detection of *Salmonella* spp. and *L. monocytogenes*. In this study, we investigated the possibility that components of the microbiota of vacuum-packed pork loins can be useful indicators of temperature deviations in an assay reproducing possible conditions encountered during the overseas transport of fresh vacuum-packed pork loins.

## 2. Materials and Methods

### 2.1. Sampling

**Figure 1:**
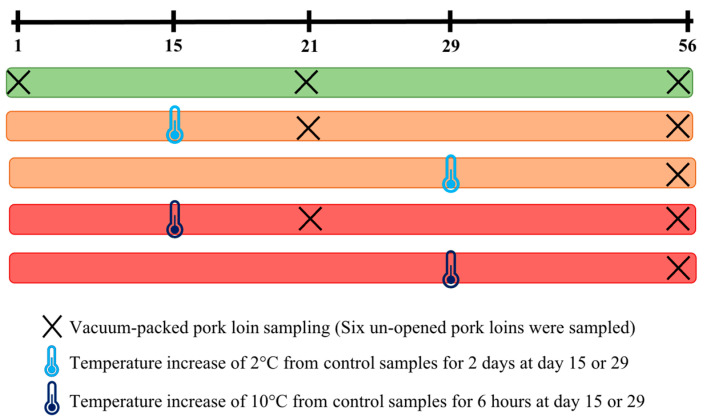
Sampling of six vacuum-packed pork loins for each condition per batch: control (stable temperature) sampled at day 1, day 21, and day 56; a temperature increase of 2 °C for 2 days at day 15, sampled at day 21 and day 56; a temperature increase of 2 °C for 2 days at day 29, sampled at day 56; a temperature increase of 10 °C for 6 h at day 15 sampled, at day 21 and day 56; a temperature increase of 10 °C for 6 h at day 29, sampled at day 56. This sampling was repeated for the 8 batches.

Fresh vacuum-packed pork loins intended to be sent overseas were collected at a swine slaughterhouse in the province of Quebec, Canada. Vacuum-packed pork loins were stored in commercial refrigerated trailers in the slaughterhouse vicinity. Trailers were kept at −1.5 °C for 56 days, a period representing the maximum time needed for fresh products to arrive and be purchased overseas. A batch of vacuum-packed pork loins was defined as products originating from animals slaughtered at the same time, i.e., during the same morning over 4 consecutive hours of processing activities. Whole packed loins were produced on Mondays or on Fridays. For the Monday samples, carcasses were cooled down for 24 h to reach a final temperature around 0 °C before packaging, while those produced on the Fridays had a 48 h cooling time. At each sampling time point, 4 batches of loins cooled for 24 h (referred to in this study as Tuesday loins) or 48 h (referred to in this study as Monday loins) before packaging were sampled. The loins were subjected to different storage temperature deviations, mimicking the defect in temperature regulation regularly encountered in the industry: (A) no deviation (control, −1.5 °C) for 56 days, (B) a temperature increase of 2 °C for 2 days at day 15, (C) a temperature increase of 2 °C for 2 days at day 29, (D) a temperature increase of 10 °C for 6 h at day 15, and (E a temperature increase of 10 °C for 6 h at day 29. Whole unopened loins were sampled at day 1 (after packaging), at day 21 (representing the time when samples would be loaded onto a boat), or at day 56 (representing the maximum period needed for the client to receive the loins overseas) (Figure 1). When temperature deviations were performed, vacuum-packed loins were moved to different trailers at the desired temperature. Temperatures were monitored using a probe located in the middle of each storage trailer, and data were collected by computer for each batch and each condition. 

For each batch (n = 8), six vacuum-packed pork loins at each sampling point (n = 9) were used, for a total of 432 vacuum-packed pork loins sampled in this study. For each sample, a vacuum-packed pork loin was opened, and a single sample consisting of a fine slice of 10 g was sterilely taken from the surface of the loin before being transferred directly into a sterile tube that was sent on ice to the laboratory for bacteriological analysis. A second sample of 10 g was also collected in the same way and was stored directly in liquid nitrogen at the slaughterhouse before being kept at −80 °C at the laboratory until DNA extractions were performed. The sampling area for each loin was randomly selected, apart from the area near the opening, which was never sampled.

### 2.2. Detection of Listeria monocytogenes and Salmonella *spp*.

#### 2.2.1. Sample Preparation

For *L. monocytogenes*, five grams of meat were transferred into a sterile filter bag (Fisher) containing 45 mL of UVM1 modified broth (Biokar diagnostics, Allonne, France). Samples were mixed for 1 min in a stomacher Smasher™ AESAP1064 (bioMérieux, St. Louis, MO, USA) and thereafter incubated at 30 °C for 48 h. After incubation, 3 mL were transferred into a 15 mL tube containing 1 mL of glycerol, and the tubes were stored at −20 °C. For *Salmonella* spp., 5 g were transferred into a sterile filter bag containing 45 mL of buffered-peptone water (ThermoFisher Scientific, Ottawa, ON, Canada). After mixing for 1 min using a stomacher, samples were incubated at 37 °C for 24 h. After incubation, 3 mL were transferred into a 15 mL conical tube containing 1 mL of glycerol, and samples were stored at −20 °C until analysis. 

#### 2.2.2. Detection

The detection of *Listeria monocytogenes* was performed as previously described by our group [36]. Briefly, thawed samples were enriched in Fraser broth (Biokar diagnostic) (24 h, 37 °C) and plated onto the selective chromogenic RAPID’*L.Mono* (Bio-Rad Laboratories Inc., Montreal, QC, Canada) agar medium. Samples were incubated for 24 h at 37 °C. The presumptive identification of *L. monocytogenes* was performed by the rhamnose fermentation test in Purple Broth Base (HiMedia Laboratories, Mumbai, India) and identification was performed by a multiplex serogrouping PCR [37]. The detection of *Salmonella* spp. was based on the methods used in previous studies [38,39]. Briefly, thawed samples were cultured on Modified Semi-Solid Rappaport-Vassiliadis Agar (MSRV) (Biokar diagnostic) as a selective enrichment step and two selective media: Brilliant Green Sulfa (BD Difco, Franklin Lakes, NJ, USA) agar and Xylose-Lysine-Desoxycholate (Biokar diagnostic) agar. The presumptive identification of two typical colonies from each culture medium was achieved using triple sugar iron agar slants (BD Difco) and urea agar slants (Statens Serum Institut, Copenhagen, Denmark).

### 2.3. 16S rRNA Gene Amplicon Sequencing

#### 2.3.1. Sample Preparation

Ten grams of meat were thawed at room temperature (for less than one hour) before being transferred into a sterile filter bag containing 40 mL of buffer (EDTA, 1 mM; Tris-Hcl, 10 mM; NaCl, 8.5 g) and mixed with a stomacher for 1 min. After that, 40 mL of the filtrate was centrifuged for 20 min at 5000 rpm at 4 °C (Sorvall legend X1R centrifuge, Thermo Scientific R, Agawam, MA, USA). The pellet was used for DNA extraction. For control samples (stable temperature) collected on day 1 and 56, DNA extraction was performed individually on the six loins of each batch, accounting for a total of 96 samples. The aim was to compare the microbiota among the eight different batches at the start and the end of the study. For the other conditions, 10 g of meat from 3 samples from the same batch were pooled to reduce analysis numbers.

#### 2.3.2. DNA Extraction, PCR Amplification, and Sequencing

DNA was extracted using the HostZERO^TM^ Microbial DNA Kit from Zymo Research, following the manufacturer’s instructions. This kit was developed to reduce the amount of contaminating host DNA and therefore enrich bacterial DNA in our samples. DNA quality and concentration were measured using a NanoDrop ND-1000 Spectrophotometer (ThermoFisher Scientific) and the Qubit 3.0 High Sensitivity broad range assay (Fisher Scientific, Ottawa, ON, Canada) on a DeNovix (Wilmington, DE, USA) fluorometer. A 291 pb fragment of the V4 region of the 16S rRNA gene was amplified by PCR using universal primers 515F_III (5′-ACACTGACGAACTGGTTCTACAAGTGCAGCMGCCGCGGTAA-3′) and 806R_III (5′-TACGGTAGCAGAGACTTGGTCTGGACTACHUGGGTWTCTAAT-3′) (Invitrogen, Thermo Fisher Scientific, Waltham, MA, USA). A 30 μL PCR reaction assessment was performed using the Platinum SuperFi PCR Master Mix (Invitrogen, Burlington, ON, Canada), as previously described [39]. The PCR program consisted of an initial denaturation at 95 °C for 5 min, followed by 25 cycles of amplification that included a denaturation step at 95 °C for 30 s, an annealing step at 55 °C for 30 s, an elongation step at 72 °C for 60 s, and a final elongation step of 10 min at 72 °C in a Mastercycler^®^Nexus PCR (Eppendorf AG, Hamburg, Germany). 

Negative control of the DNA extraction (H20), as well as a PCR negative (H20) and positive control (ZymoBIOMICS Microbial Community DNA Standard, Zymo Research, Irvine, CA, USA) was included. The PCR positive control contained DNA from eight known bacterial genus with different 16S rRNA gene abundance (18.4% *Lactobacillus*, 17.4% *Bacillus*, 15.5% *Staphylococcus*, 14.1% *Listeria*, 10.4% *Salmonella*, 10.1% *Escherichia*, 9.9% *Enterococcus*, and 4.2% *Pseudomonas*). PCR amplification from samples and positive controls, as well as the absence of amplification from the negative controls, were confirmed by electrophoresis on a 1.5% agarose gel. The purification, barcoding, and sequencing were performed on an Illumina MiSeq 250 paired-end sequencing at Genome Québec Innovation Centre, Montréal, QC, Canada. 

#### 2.3.3. Sequencing Data Processing

The cleaning and the analyzing of the sequences were completed using Mothur [40] version 1.43 following the MiSeq standard operational procedure (https://mothur.org/wiki/miseq_sop/, accessed on 31 July 2021), as previously described [39]. Forward and reverse reads were merged into contigs for each sample. Sequences that contained ambiguities were removed, and identical sequence were merged. The unique sequences were aligned using the SILVA 132 reference database formatted for Mothur (https://www.mothur.org/wiki/Silva_reference_files, accessed on 31 July 2021). Sequences were pre-cluster using the Deblur algorithm and chimeras were removed using VSEARCH [41]. Sequences identified as other that bacteria were removed. The remaining sequences were clustered into operational taxonomic units (OTUs) using a unique method yielding a threshold of 100% sequence identity. In this study, the Amplicon Sequence Variant approach (ASV) was used for analysis. Final taxonomic grouping was assigned to these ASVs using the Ribosomal Database Project (RDP) trainset 16 database formatted for Mothur (https://www.mothur.org/wiki/RDP_reference_files, accessed on 31 July 2020).

#### 2.3.4. Sequencing and Statistical Analysis

Alpha and beta diversity analyses were performed using RStudio (version 1.4.1103), according to the standard operating procedure (SOP) used in a previous study [39]. Data were first rarefied to the lowest number of sequences found in a sample to minimize the impact of uneven sequencing depth. Alpha diversity indexes (Observed ASV, Shannon and Inverse Simpson) were calculated using the estimate_richness function from the R package “Phyloseq” [42], and the Kruskal–Wallis test was performed to compare the alpha diversity measures between conditions. Beta diversity was assessed using the Bray–Curtis and Jaccard distance metrics, based on the relative abundance and on the presence/absence at the genus level, respectively, and visualized using 2D non-metric multidimensional scaling (NMDS). Permutational analysis of molecule variance (PERMANOVA) were conducted using the ADONIS function from package “vegan” [43] for the analysis of the microbiota structure and assessed statistical variations between the different conditions. Alpha and beta diversity analysis were performed to investigate the following differences: between different time points; between batches at day 1 and day 56; and between each group at day 1, 21, and 56. Alpha and beta diversity analysis were also conducted to compare samples based on the production day (Monday vs. Tuesday).

## 3. Results

### 3.1. Pathogen Detection

No sample was found positive for *L. monocytogenes*. Three samples out of the 432 tested were positive for the presence of *Salmonella* spp.: one loin surface sampled from batch #3 and two samples recovered from batch #4, all from the control samples (no temperature deviation) at day 1.

### 3.2. Sequencing Data

A total of 18,647,640 sequences were generated from the analysis of the samples collected. After cleaning and processing all the reads—a total of 12,277,943 sequences—were retained and were assigned to 27 phyla, 69 classes, 129 orders, 279 families, and 821 genera. The lowest number of sequences found was 10,176. A mean of 59,153 sequences was obtained per sample, and a total of 18,961 ASV was detected. The positive PCR control was composed of 18.1% *Bacillus*, 14.7% *Lactobacillus*, 14.6% *Staphylococcus*, 12.8% *Escherichia/Shigella*, 12% *Salmonella*, 12.3% *Listeria*, 9.5% *Pseudomonas*, and 6% *Enterococcus*, a composition close to the manufacturer product description. A total of 64 and 100 sequences were obtained for the negative controls of the DNA extraction and the PCR steps, respectively. 

### 3.3. Evolution of the Microbiota of the Control Vacuum-Packed Loins

#### 3.3.1. Bacterial Composition 

**Figure 2:**
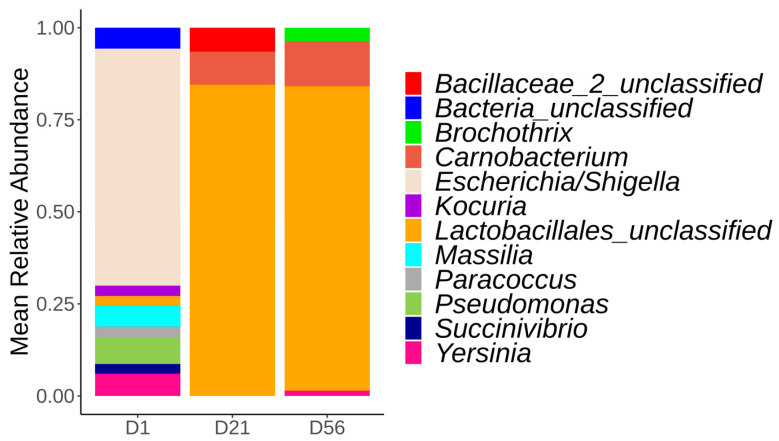
Relative abundance of the major bacterial genera identified from the vacuum-packed pork loin surface microbiota at day 1, 21, and 56. Only bacterial genera representing at least 2% of the total reads are shown.

The surface microbiota of control loins (no temperature deviation) evolved in time (Figure 2). At day 1, the vacuum-packed pork loin surface microbiota was dominated by the *Escherichia/Shigella* (58%) genus, followed by *Pseudomonas* (14%) and *Paracoccus* (5%). At day 21, there was a clear shift in the surface microbiota, moving from a dominance of *Escherichia/Shigella* to the *Lactobacillales_unclassified*, which represented over 80% of the microbiota composition. *Carnobacterium* (12%) and *Bacillaceae_unclassified* (8%) genera were also present at day 21. At day 56, mainly *Lactobacillales_unclassified* (75%), *Carnobacterium* (16%), *and Brochothrix* (4%) genera formed the surface microbiota of the analyzed loins. Since the *Lactobacillales_unclassified* was composed of a single ASV, the sequence was analyzed with the online Basic Local Alignment Search Tool (BLAST) in an attempt to refine this ASV taxonomy. *Lactobacillales_unclassified* ASV was identified as *Lactobacillus* spp. (query cover of 100%). Further inspection of the sequencing results revealed that this ASV sequence was present in samples at day 1, but in low relative abundance (3%). 

An ASV assigned to *Yersinia*, a potential foodborne pathogen, was also observed. This same ASV was found at day 1 and at day 56 and was identified as *Yersinia enterocolitica* (query cover of 100%) using online BLAST. At day 1, 20.8% (10/48) of the control samples contained *Yersinia* ASV, with a mean relative abundance of 5.9%. At least one sample from each batch contained this ASV, except batch #1 and batch #2, from which it was not identified. At day 56, 37.5% (18/48) of the samples from the control group contained *Yersinia* ASV (with a mean relative abundance of 0.6%), while 45.3% (29/64) of the samples submitted to a temperature increase of 2 °C or 10 °C at day 15 or 29 were positive (with a mean relative abundance of 2.3%). 

#### 3.3.2. Alpha and Beta Diversities 

**Figure 3:**
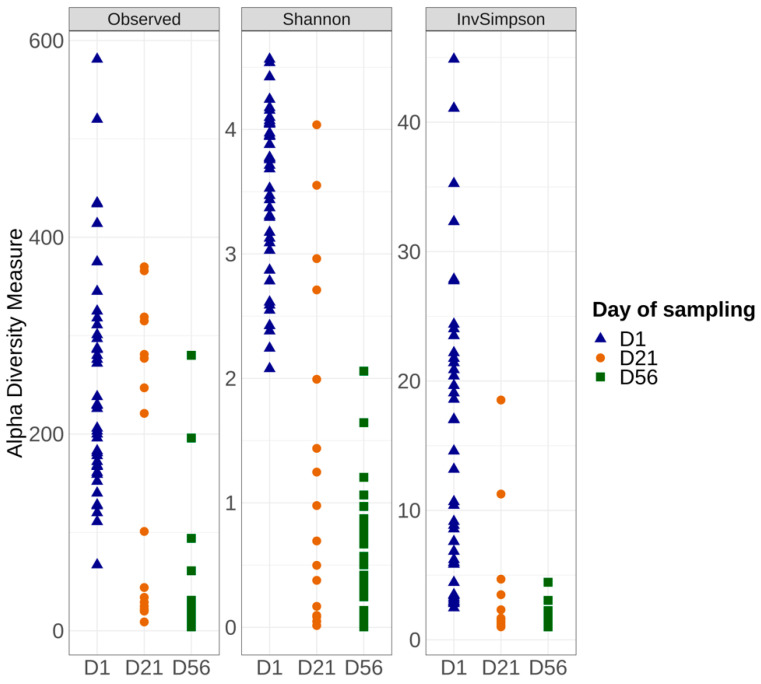
Alpha diversity measures among the control samples at day 1, 21, and 56 using Observed, Shannon, and Inverse Simpson indices.

The microbiota alpha and beta diversity of the control samples were compared between production days at each time point (Monday vs. Friday), and no significant differences could be observed (Kruskal–Wallis test *p* > 0.05). This factor was therefore not taken into consideration for downstream analyses. Alpha diversity analysis, which describes the bacterial richness and distribution within a sample, was used to compare the surface of vacuum pork loin microbiota for the control samples at days 1, 21, and 56 as shown in Figure 3. Significant differences were noted for the Observed, Shannon, and Inverse Simpson indices. Samples from day 1 showed alpha diversity indices significantly higher compared to the day 21 and day 56 samples.

**Figure 4:**
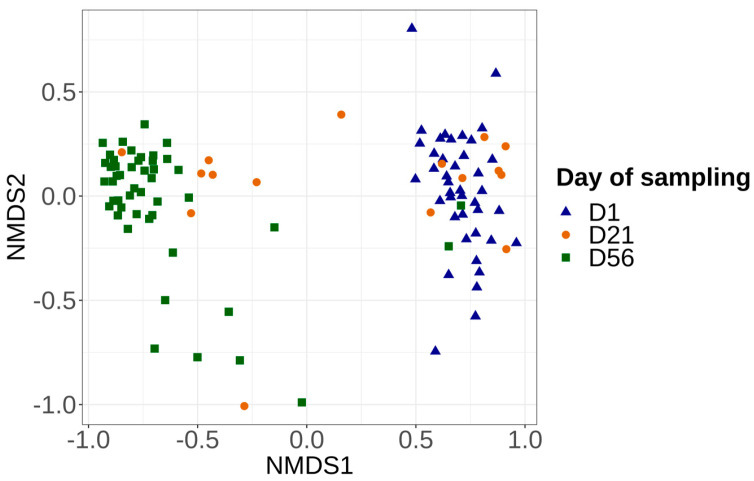
Non-metric multidimensional scaling plot (NMDS) illustrating beta diversity, calculated with the Bray–Curtis index according to the day of sampling (D1, D21, D56). Significant differences were found in the vacuum-packed surface microbiota between day 1 and 21, as well as day 1 and 56. No significant difference between day 21 and 56 was seen.

Bray–Curtis and Jaccard distance matrix results showed that the structure and membership of the bacterial community was significantly different (PERMANOVA *p* > 0.05) between the control samples (always at −1.5 °C) collected on days 1, 21, and 56. Overall, control samples at day 1 and 56 clearly clustered into two different groups (Figure 4), while samples at day 21 were distributed in these two groups. Two-by-two comparisons revealed a significant difference in the microbiota structure between samples collected on day 1 and day 21, as well as between the day 1 and day 56 samples. For samples collected on day 21, a group of samples was close to the results of day 1; these samples were from batch numbers 1, 2, 3, 4, and 8. Another group of day 21 samples was grouped with day 56, and these samples were from batches 4, 5, 6, and 7 (Figure S1).

### 3.4. Impact of Production Batch on the Microbiota of Control Samples at Day 1 and Day 56

**Figure 5:**
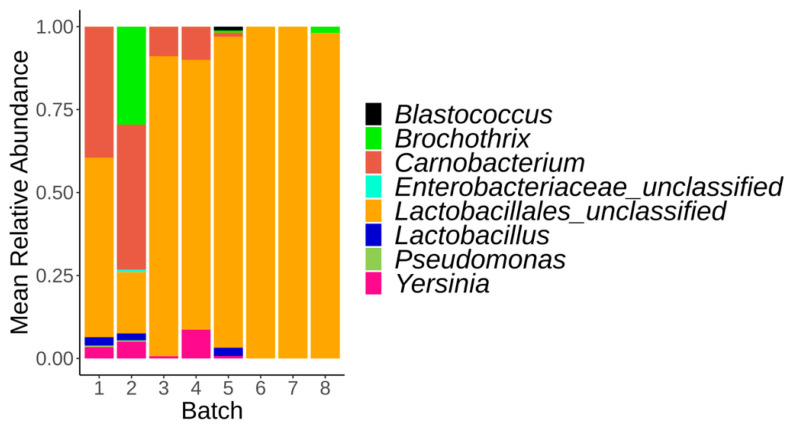
Relative abundance of the major bacterial genera identified on the vacuum-packed pork loin surface microbiota at day 56 for each batch. Only the bacterial genera representing at least 2% of the total reads are shown.

Alpha and beta diversity were first compared between control samples (constant temperature at −1.5 °C) collected on day 1 between the eight batches. No significant difference could be observed at day 1 for both alpha (Figure S2) and beta diversity (Figure S1). The same analysis was repeated at day 56. For both alpha and beta diversity, the results showed a significant difference between the 8 batches (Figures S1 and S2). Observed and Shannon index values were higher for batch number 1 and 2 compared to the six other batches. For beta diversity, two-by-two comparison between the eight batches revealed that samples from batch 1 were significantly different from those collected from batches 6, 7, and 8, while samples from batch 2 were different from those analyzed for batches 3, 4, and 5 (Figure S2). The main bacterial genera observed at day 56 for samples from batches 1 and 2 were *Carnobacterium* and *Brochothrix*, respectively, while batches 3, 4, 5, 6, 7, and 8 were dominated by the *Lactobacillales_unclassified* genus (Figure 5).

### 3.5. Impact of Temperature Deviations on the Vacuum-Packed Pork Loin Surface Microbiota at Day 56 

**Figure 6:**
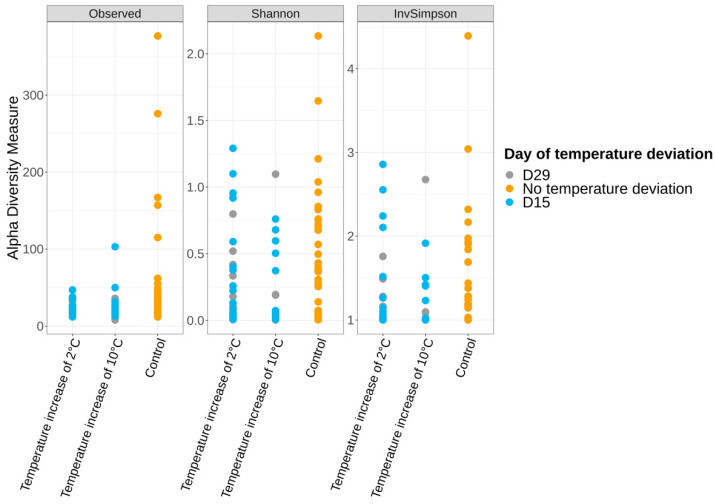
Alpha diversity measures among the control samples and samples that were subjected to a temperature increase of 2 °C or 10 °C, carried out at day 15 and 29 and sampled at day 56.

**Figure 7:**
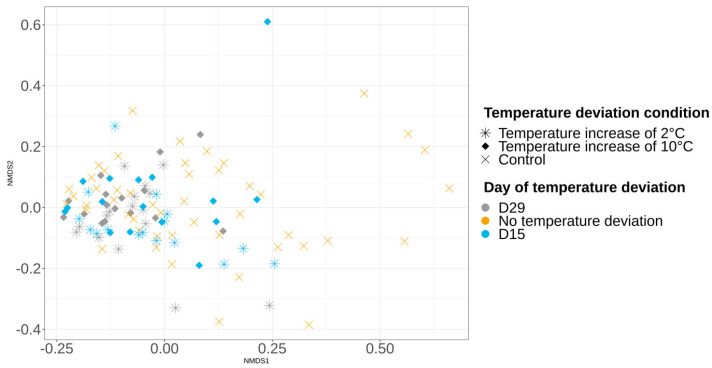
Non-metric multidimensional scaling plot (NMDS) illustrating microbiota beta diversity, calculated with the Bray–Curtis index, comparing control samples to samples that were subjected to a temperature increase of 2 °C or 10 °C, carried out at day 15 and 29, and sampled at day 56.

At day 56, the alpha and beta diversity analysis showed that the richness, structure, and membership of the bacterial community were similar (Kruskal–Wallis and PERMANOVA *p* > 0.05) between the control samples and samples that were subjected to a temperature increase of 2 °C or 10 °C, carried out at day 15 (Figure 6 and Figure 7). This same observation was made for the comparison between control samples and samples that were subjected to a temperature increase of 2 °C or 10 °C, carried out at day 29 (Figure 6 and Figure 7). The same observations were observed for samples collected on day 21. 

## 4. Discussion

In this study, the surface microbiota of vacuum-packed pork loins was analyzed by high-throughput sequencing and culture-dependent methods for pathogens detection in order to determine the evolution of the microbiota over a period of several weeks under refrigerated conditions and with an early or late transient temperature deviation. The objective of this study was to characterize the impact of temperature deviations on the microbiota of vacuum-packed pork loins according to different production batches.

In the present study, the non-detection of *L. monocytogenes* can be explained by an absent or very low initial contamination and/or by the absence of growth during storage, for all conditions tested. In a previous study conducted in the same facility, it was observed that conveyors on which loins travel during processing were never contaminated by *L. monocytogenes*, while conveyors associated with Boston or shoulder meat pieces were contaminated by the pathogen [36]. As a comparison, Saraiva et al. [44] observed that *L. monocytogenes* survived better in refrigerated vacuum-packed meat compared to fresh air-packed products. Previous studies also reported a prevalence ranging from 15.9% to 33.3% for *L. monocytogenes* in fresh meat [45,46]. As for *Salmonella* spp., few positive samples were detected, and only at day 1. The absence of *Salmonella* spp. at day 56 reinforces previous work results that suggest a decrease in *Salmonella* spp. population on vacuum-packed pork meat with storage duration [26,47].

Microbiota analysis using high-throughput sequencing allowed us to reveal the presence of a *Yersinia* ASV that was further identified as *Yersinia enterocolitica*. It is known that pigs are a reservoir of pathogenic *Y. enterocolitica*, a bacterium responsible for human foodborne illnesses [48]. Moreover, *Y. enterocolitica* is naturally found in the intestinal content and tonsils [49] of pigs, and it is reported that evisceration or the removal of tonsils is a major source of contamination for carcasses during the slaughtering process [48]. The prevalence of *Y. enterocolitica* on pork carcasses reported in previous studies ranged from 2% to 39.7% [23,50,51,52]. In this study, we observed that the *Yersinia* genus was more frequently detected on samples collected on day 56 as compared to day 1. It is known that *Y. enterocolitica* can grow at refrigerated temperatures in vacuum-packed meat products [53]. 

The microbial composition, diversity, and structure of the surface microbiota of vacuum-packed pork loins were different between days 1 and 21 (when a shift from aerobic to anaerobic bacteria populations was observed) and between day 1 and day 56. These results are in line with those of other authors [15,54], who found that the bacterial community was more diverse at the beginning of storage and tended to decrease over time. As observed by Zhao et al. [15], it is important to notice that the decrease in the diversity of pork vacuum-packed microbiota during storage is not to be interpreted as a decrease of bacterial contamination, as the bacterial count continued to increase.

At day 1, *Escherichia/Shigella* genera was the most abundant bacterial population for each batch, followed by *Pseudomonas*. *Escherichia/Shigella* (*Enterobacteriaceae* family) are coliform bacteria considered indictors of fecal contamination. Their presence usually suggests insufficient sanitary conditions or possible cross-contamination events before the packaging and storage stages [55]. Moreover, we observed that the relative abundance of *Escherichia/Shigella* was greater than *Pseudomonas*, while *Pseudomonas* was the most abundant genus found in fresh meat or at the beginning of the packaging step and is a major spoilage microorganism of fresh meats associated with environmental contamination [5,18].

On sampling days 21 and 56, *Lactobacillales_unclassified* genus was the main constituent of the vacuum-packed pork loin surface microbiota stored at −1.5 °C. The order of Lactobacillales includes families that are part of the LAB. In addition to LAB, *Brochothrix thermosphacta* and *Carnobacterium* were also reported as the major component of the microbiota in chilled vacuum-packed pork under extended storage (for up to 21 days) [15,31,33]. In the current study, it is important to note that when the vacuum-packed pork loins were opened, no off-odor, discoloration of the meat, or packaging default were observed in any pork loin sampled, suggesting that the level of contamination from LAB was below spoiling levels.

In the current study, no differences were recorded for the surface microbiota of pork loins at the first day of the vacuum packaging between batches. Meat processing steps, until packaging, may have standardized the surface carcass microbiota of pigs, as observed in a previous study conducted in the same slaughterhouse [39]. However, in the current study, the microbiota diversity had evolved in these same batches of pigs at day 56. The diversity and structure of the microbiota of two batches were significantly different compared to the six other batches, with *Carnobacterium* being predominate in batch #1 and #2. Other studies [20,56] have reported the dominance of *Carnobacterium* spp. at the end of a long storage period (at −0.5 °C for 3 months) in vacuum-packaged meat products. In the present study, the microbiota difference observed between batches at day 56 was not anticipated, as all vacuum-packed pork loins had been stored in the same conditions, and no difference between batches could be observed at the start of the study. During the 56-day storage at −1.5 °C, bacteria may not have grown uniformly between batches due to a combination of factors, such as concentration of nutriments (glucose vs. amino acid), the presence of low molecular weight compounds in meat exudates, pH, packaging atmosphere composition, and environmental production factors (temperature, humidity) [57,58]. Moreover, in the present study, the differences in meat quality and aging might also be responsible for the differences observed between the batches at day 56. Faucitano et al. [59] observed that bacterial counts of total aerobic mesophilic and LAB on fresh vacuum-packed pork loins were similar at the beginning of storage, but evolved differently after 35 days, according to different meat quality classes. For example, it was reported that dark, firm, dry meat presented higher pH and was the meat quality class most susceptible to spoilage. Another possibility is that the initial bacterial loads may have been different between batches at day 1, although these might have been too slight to be observed by sequencing. These small, undetected differences might have been amplified over the 56 days of storage. The unknown total bacterial load on sampling day 1 is a limiting factor of our study. Complementary data of bacterial enumeration of mesophilic aerobic bacteria, total coliform, *Escherichia coli*, and anaerobic lactic bacteria was given by the slaughterhouse for other loin samples taken at day 56. We observed that different samples within a same batch showed differences between LAB bacterial counts ranging from 10^4^ to 10^7^ log UFC/g. This suggests the hypothesis that diversity differences observed at day 56 between the batches might be due to different bacterial loads. Indeed, Zhao et al. [12] observed that the diversity of spoiled (with bacterial concentration of bacteria aerobic total > 6 log_10_ CFU/g) pork meat was significantly lower than fresh (not spoiled) meat stored at −2 °C or 4 °C. 

The increase in storage temperature (increase of 2 °C for 2 days and increase of 10 °C for 6 h) at day 15 or day 29, which simulated a defect in temperature regulation, had no recordable influence on the microbiota composition and diversity of vacuum-packed pork loins. On fresh pork meat not under vacuum packaging, Zhao et al. [12] observed that the diversity of spoiled pork meat stored at 10 °C during 72 h was significantly higher than that of fresh pork. In vacuum-packed beef meat, Yang et al. [60] observed that the microbiota was dominated by *Carnobacterium* during 150 days of storage at 4 °C, 2 °C and −1 °C, but only a positive association between *Carnobacterium* and meat stored at 2 °C and −1 °C was found. Moreover, *Lactobacillus* was predominant at the end of storage at 2 °C, as well as at −1 °C, while the abundance of *Lactococcus* increased with storage time at 4 °C. In our study, the absence of an impact of the different temperature deviations on the microbiota could be explained by the fact that the increase in the temperature was not high and/or not long enough to have a significant effect on the meat microbiota. Another possibility is that there was a transient modification of the microbiota that was not covered by the used sampling time points (day 21 and 56). During 56 days of storage, specific members of the microbiota of vacuum-packed pork loins could not be used as biomarkers of temperature deviations.

## 5. Conclusions

The present results are specific to this study, which was conducted in a single slaughterhouse on one specific pork product. To our knowledge, this is the first time that a study has described the overall microbiota of vacuum-packed pork loins according to different temperature deviations and production batches in a design corresponding to the transport of fresh meat products overseas. Results showed that the surface microbiota of pork loins at the first day of the vacuum packaging appear similar between batches, but had differently evolved between by the end of storage. Our results showed that a slight deviation in temperature, followed by a return to normal, was not sufficient to affect the final bacterial composition and diversity of vacuum-packed pork loins. This study showed that high throughput sequencing is a useful tool that may identify unsuspected problems in a slaughterhouse, such as differences between batches after storage or the presence of unexpected pathogens such as *Yersinia*.

## Data Availability

The sequencing data were openly available in the NBCI Sequence Read Archive under accession number PRJNA868338 at https://www.ncbi.nlm.nih.gov/bioproject/PRJNA868338/ (accessed on 10 August 2022).

## Supplementary Materials

The following supporting information can be downloaded at: https://www.mdpi.com/article/10.3390/foods12081695/s1, Figure S1: Non-metric multidimensional scaling plot (NMDS) illustrating microbiota beta diversity, calculated with the Bray–Curtis method, according to batch and day of sampling; Figure S2: Alpha diversity measures among the control samples at day 1 and 56 for the eight batches of the surface of vacuum pork loins sampled using Observed, Shannon, and Inverse Simpson indices.
