# Supplementary material for: Slight Temperature Deviation during a 56-Day Storage Period Does Not Affect the Microbiota of Fresh Vacuum-Packed Pork Loins
Source: Foods. 2023 Apr 19;12(8):1695. doi: 10.3390/foods12081695 (PMC10138144; doi:10.3390/foods12081695)
Supplement: Supplementary file 1 [file foods-12-01695-s001.zip › foods-2339275-supplementary.pdf]

Supplementary Material

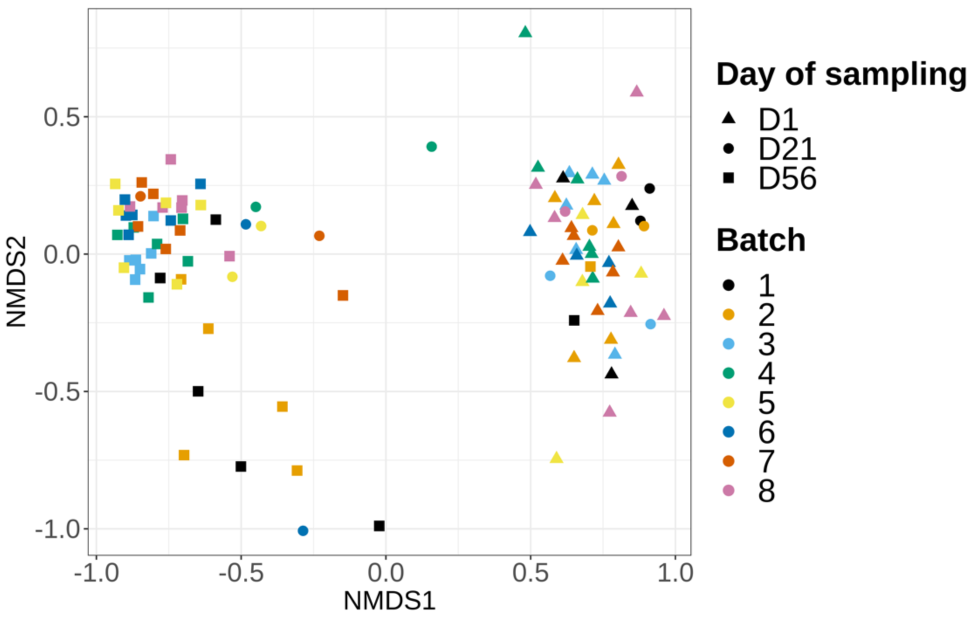

Figure S1. Non-metric multidimensional scaling plot (NMDS) illustrating microbiota beta diversity, calculated with the Bray-Curtis, according to batch and day of sampling.

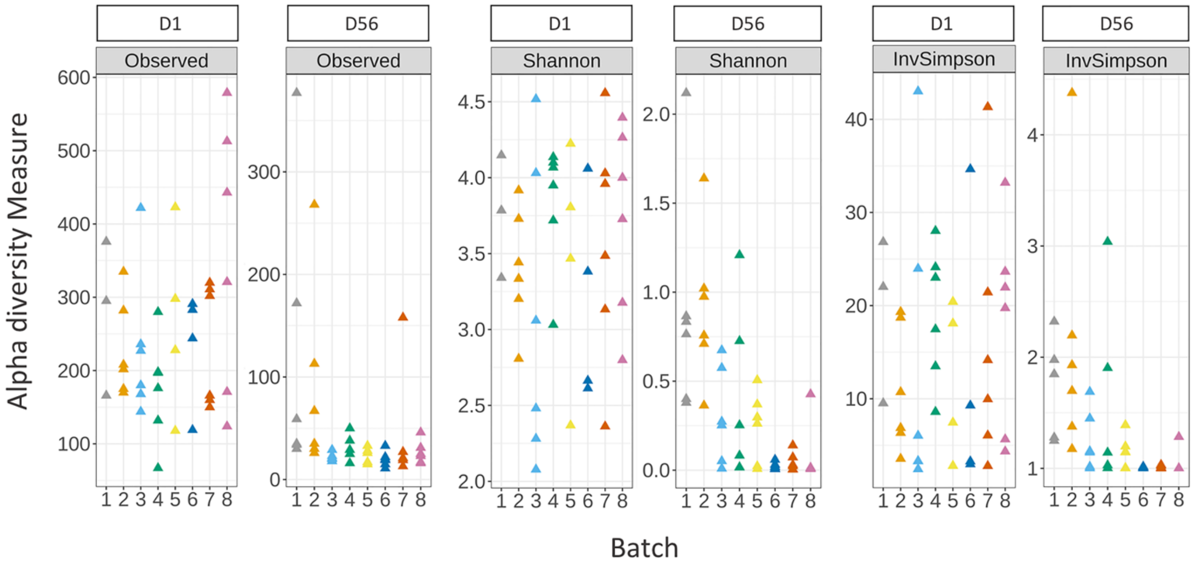

Figure S2. Alpha diversity measures among the control samples at day 1 and 56 for the 8 batches of the surface of vacuum pork loin sampled using Observed, Shannon, and Inverse Simpson indices.
